# Progress in Achieving LDL Cholesterol Target Levels in a High-Risk Patient Population in Slovakia

**DOI:** 10.3390/diagnostics16131980

**Published:** 2026-06-25

**Authors:** Stefan Toth, Lukas Olsavsky, Pavol Fulop, Mariana Dvoroznakova, Martin Sevcik, Natalia Vanova, Viliam Weis

**Affiliations:** 1 Department of Histology and Embryology, Faculty of Medicine, Pavol Jozef Safarik University, 040 11 Kosice, Slovakia; stefan.toth@upjs.sk (S.T.); viliam.weis@unlp.sk (V.W.); 2 Cardiology Outpatient Clinic, Kardiocomp, s.r.o., 040 01 Kosice, Slovakia; 3 Faculty Hospital Agel Kosice-Saca, 040 15 Kosice, Slovakia; olsavsky.lukas@gmail.com; 4 East Slovak Institute of Cardiovascular Diseases, 040 01 Kosice, Slovakia; pfulop@vusch.sk (P.F.); mdvoroznakova@vusch.sk (M.D.); 5 Faculty Hospital of Jan Adam Rayman, 080 01 Presov, Slovakia; sevmart11@gmail.com

**Keywords:** dyslipidemia, cardiovascular risk, target values, LDL cholesterol

## Abstract

**Background/Objectives:** The management of dyslipidaemia in Slovakia has undergone significant changes in recent years, particularly through the relaxation of prescription restrictions for existing medications and the introduction of new innovative molecules. Achieving target levels of LDL cholesterol (LDL-C) plays a key role in preventing the onset and progression of atherosclerosis-related cardiovascular (CV) diseases. The aim of this study was to analyse how these changes have affected the effectiveness of reaching target LDL-C levels in patients at very high CV risk. **Methods:** This project was conducted as a retrospective analysis of anonymised LDL-C values from 2020 to 2023 using data from a collaborating nationwide laboratory. Patients included were those diagnosed with acute coronary syndrome (ACS), stroke, and, more generally, those with high and very high CV risk. Target LDL-C values were assessed based on the 2019 ESC/EAS guidelines. **Results:** A total of 363,020 LDL-C test records from 115,950 patients were evaluated over the four-year study period. Among patients diagnosed with ACS, 2.2–5% achieved target LDL-C levels in the respective years of observation 2020–2023. As many as 6.5–7.4% had LDL-C levels ≥ 4.9 mmol/L. For patients with stroke, only 4–6.6% reached target LDL-C levels, while 5.6–6.7% had levels ≥ 4.9 mmol/L. In the group with very high CV risk, only 1.7–3% achieved target levels, and 7.5–8.7% had extremely high LDL-C levels ≥ 4.9 mmol/L. Despite these modest improvements, over 93.4% of patients in the highest-performing subgroup failed to reach the absolute guideline target threshold in 2023. **Conclusions:** While the lifting of prescription constraints and the introduction of innovative treatments correlates with a doubling of absolute target attainment and a contraction of extreme hypercholesterolemia, overall control remains critically low in Slovakia. Systematic, protocol-driven combination regimens and intensive follow-up are urgently needed.

## 1. Introduction

- Despite significant regulatory changes in Slovakia—including the lifting of prescription restrictions for statins/ezetimibe and the introduction of inclisiran and bempedoic acid—the vast majority of very high cardiovascular risk patients still fail to reach LDL-C targets, with over 93% missing guideline-recommended levels even in 2023
- Among post-ACS patients, only 2.2–5.0% achieved the target LDL-C of <1.4 mmol/L across 2020–2023; similarly low rates were seen in post-stroke patients (4.0–6.6%) and the broader very high CV risk group (1.7–3.0%)
- A positive trend emerged in 2022–2023, with target attainment roughly doubling compared to the start of the observation period, likely driven by policy changes and the rollout of novel lipid-lowering therapies
- Lipid monitoring frequency remains a serious concern—only 19% of ACS patients and 11% of stroke patients had their lipid profiles checked annually, pointing to significant gaps in outpatient follow-up and long-term disease management
- Slovakia lags well behind the European average in LDL-C control, with comparator studies from countries like the Netherlands, Greece, and Spain reporting target attainment rates several times higher, underscoring the need for intensified patient monitoring, cardiac rehabilitation, and physician education.

The development and progression of atherosclerotic cardiovascular diseases (ASCVD) are strongly associated with LDL cholesterol (LDL-C) concentrations, as well as the duration over which elevated levels influence atherosclerotic plaque progression [1]. Genetic (Mendelian) studies that laid the foundations for PCSK9 research, together with large observational statin studies and results from recent clinical trials with PCSK9 inhibitors and meta-analyses, unequivocally confirm that reducing LDL-C levels significantly reduces the risk of the development and progression of atherosclerotic disease [2]. Furthermore, long-term maintenance of low, even extremely low, LDL-C levels is safe and well tolerated, potentially leading to mild but statistically significant regression of atherosclerotic plaques [3].

In our previous SlovakLipid study, we analysed data from 2017 to 2019, examining the attainment of target LDL-C levels in patients at very high cardiovascular (CV) risk [4]. The results showed that the majority of patients (91–95%) did not achieve the target values set by the 2016 ESC/EAS guidelines [5]. When applying the updated 2019 recommendations—relevant for the last year of the study period—this proportion was even higher (97–99%) [6].

Slovakia is among countries with very high cardiovascular risk [7]. Despite advances in acute hospital care, including progress in imaging diagnostics and interventional cardiology, the decline in CV mortality is minimal, pointing to poor risk factor control after hospital discharge, the absence of intensified outpatient follow-up for these patients, a lack of cardiac rehabilitation programmes, and a degree of “resistance” among physicians to prescribe lipid-lowering agents [8,9]. Effective control of the lipid profile—particularly LDL-C levels—remains one of the key determinants of successful prevention. In 2022–2024, significant changes were made in Slovakia regarding prescription and indication restrictions for available lipid-lowering agents (atorvastatin, rosuvastatin, ezetimibe and their combinations). At the same time, new drugs reimbursed from public health insurance were fully integrated into clinical practice—the PCSK9 inhibitor inclisiran and the ACL inhibitor bempedoic acid—against the backdrop of the new 2019 EAS/ESC guidelines, which, based on new studies, tightened LDL-C target values across the risk spectrum and thereby further worsened the already poor attainment of target LDL-C levels in patients [6].

The aim of this study was to retrospectively analyse anonymised laboratory data on LDL-C values in patients classified as high- and very high-risk, with a diagnosis of acute coronary syndrome (ACS), stroke, or documented ASCVD (high-risk and very high-risk groups). We also aimed to determine the proportion of patients achieving LDL-C target values according to current ESC/EAS recommendations, and to analyse how changes in the availability and prescription rules for lipid-lowering therapy have translated into real clinical practice.

## 2. Methods

### 2.1. Study Design

The present analysis follows the same general framework as the original SlovakLipid study, using a retrospective design based on large-scale, anonymised LDL-C laboratory data from patients receiving care across outpatient and inpatient facilities throughout Slovakia [4]. Laboratory records were sourced from a nationwide private laboratory group. The observation window spanned four consecutive years from 2020 to 2023, a period during which the 2019 ESC/EAS dyslipidaemia guidelines were in force [6]. Because the database was entirely anonymized at the point of retrieval and contained no direct personal or demographic identifiers, and since all patients had previously provided written general informed consent at the time of sample collection permitting the use of de-identified data for clinical research, a formal ethics committee approval waiver was granted in accordance with Slovak national healthcare legislation on retrospective database research.

Eligible patients were first identified on the basis of ICD-10 diagnosis codes consistent with very high cardiovascular risk. Once identified, all LDL-C measurements recorded for these patients between 2020 and 2023 were retrieved, irrespective of the diagnosis listed at the time of each individual measurement. Serum LDL-C concentrations across all collaborating collection sites were primarily calculated using the standard Friedewald formula. However, to preserve analytical accuracy, if a patient’s total serum triglyceride concentration exceeded 4.5 mmol/L (approx. 400 mg/dL), the laboratory automatically bypassed calculation and performed a direct enzymatic assay measurement. All central processing facilities involved in the analysis adhered strictly to continuous internal quality control protocols and participated in mandated external quality assessment (EQA) schemes. Precise patient fasting status prior to venipuncture could not be strictly mandated or verified due to the real-world, retrospective registry structure of the database.

To allow more granular tracking of lipid control, patients were assigned to one of eight LDL-C intervals: <1.4 mmol/L (<55 mg/dL); 1.4–1.8 mmol/L (55–70 mg/dL); 1.8–2.6 mmol/L (70–100 mg/dL); 2.6–3.0 mmol/L (100–115 mg/dL); 3.0–3.5 mmol/L (115–135 mg/dL); 3.5–4.0 mmol/L (135–155 mg/dL); 4.0–4.9 mmol/L (155–190 mg/dL); and ≥4.9 mmol/L (≥190 mg/dL).

### 2.2. Eligibility Criteria

The observation window spanned four full calendar years from 1 January 2020 to 31 December 2023, during which the 2019 ESC/EAS dyslipidaemia guidelines were active [6]. Eligible individuals were identified on the basis of International Classification of Diseases, 10th Revision (ICD-10) diagnosis codes recorded on laboratory request forms submitted by referring physicians. Eligible patients were stratified into three distinct but overlapping clinical groups:

**Group 1 (Acute Coronary Syndrome (ACS)):** Patients with a documented history of acute coronary events, defined by ICD-10 codes I20.0 (unstable angina), I21 (acute myocardial infarction), and I22 (subsequent myocardial infarction).

**Group 2 (Ischemic Stroke):** Patients with a definitive history of acute ischemic cerebrovascular events, explicitly restricted to ICD-10 codes I63.5 (cerebral infarction due to unspecified occlusion or stenosis of cerebral arteries), I63.8 (other cerebral infarction), and I63.9 (cerebral infarction, unspecified). This strict diagnostic boundary was selected to minimise false-positive misclassification and exclude non-ischemic or unconfirmed conditions (e.g., precerebral arterial occlusions without documented tissue infarction under codes I63.0–I63.4), thereby ensuring an accurate secondary prevention stroke cohort.

**Group 3 (Broader High- and Very High-Risk Population):** A comprehensive secondary and primary prevention cohort that includes all patients from Groups 1 and 2, as well as individuals with documented stable chronic coronary syndromes (ICD code I25) or peripheral arterial atherosclerosis (ICD code I70).

It must be acknowledged that because individual longitudinal clinical histories and granular phenotype details were unavailable in the laboratory registry, Group 3 represents an aggregate population managed under the assumption of high- or very high-risk. To maintain an objective, guideline-aligned framework, we evaluated the ‘achievement of the absolute LDL-C threshold recommended for very high-risk patients’ (<1.4 mmol/L) across the entire cohort, recognising that for a minor high-risk subgroup the guideline-defined absolute target is technically <1.8 mmol/L. Because untreated baseline lipid levels were unrecorded, we could not evaluate the relative ≥50% reduction component of guideline targets, and thus report strictly on absolute threshold achievement.

Once an eligible patient was identified via an ICD-10 code, all subsequent longitudinal LDL-C records between 2020 and 2023 were extracted, independent of the diagnosis listed on each individual sub-request. Where a patient had multiple measurements within a calendar year, the highest annual value was selected as the primary metric to provide a conservative, stringent evaluation of worst annual lipid control and maximal residual risk. To ensure comparability with other international registries, a secondary sensitivity analysis was performed utilising each patient’s final (most recent) recorded measurement per calendar year. If a patient met the criteria for multiple categories (e.g., experiencing an ACS event followed by a stroke), their data were counted within both specific disease trackers, while contributing uniquely to the overall Group 3 denominator. To monitor granular longitudinal shifts, values were distributed across eight harmonised, high-contrast lipid categories spanning from <1.4 mmol/L up to ≥4.9 mmol/L.

### 2.3. Statistical Analysis

Statistical analyses were executed using IBM SPSS Statistics version 27.0 (IBM Corp., Armonk, NY, USA) and R version 4.2.1 (R Foundation for Statistical Computing, Vienna, Austria). Categorical tracking of annual target proportions was validated via the Cochran–Armitage trend test (Z statistic). To evaluate changes in continuous mean LDL-C levels over the four-year study period while accounting for the unbalanced panel nature of the registry (as a substantial proportion of patients did not have blood drawn in all four consecutive years), a Linear Mixed-Effects Model (LMM) was implemented rather than a traditional repeated-measures ANOVA. Fixed effects included time (years as a continuous or categorical factor), patient sex, and their interaction, while unique patient identification codes were modelled as random effects to control for intra-individual correlation. Type III F-statistics, corresponding *p*-values, and partial omega-squared (ω^2^) or fixed-effect estimates are reported to provide rigorous inferential robustness. Statistical significance was predefined as a two-sided *p*-value < 0.05.

## 3. Results

### 3.1. Sample Size and Patients’ Characteristics

Analysis of the four-year dataset yielded a total of 363,020 LDL-C test records from 115,950 patients. Patients were divided into groups based on type of CV disease according to the protocol (Table 1). For statistical robustness, a Two-Way Linear Mixed-Effects Model was fitted for each group to rigorously assess continuous log-transformed LDL-C concentrations as a function of time, sex, and their interaction. This allowed us to report definitive F-statistics, *p*-values, and effect sizes (Table 2).

### 3.2. Detailed Evaluation of Group 1 (ACS)

**Figure 1:**
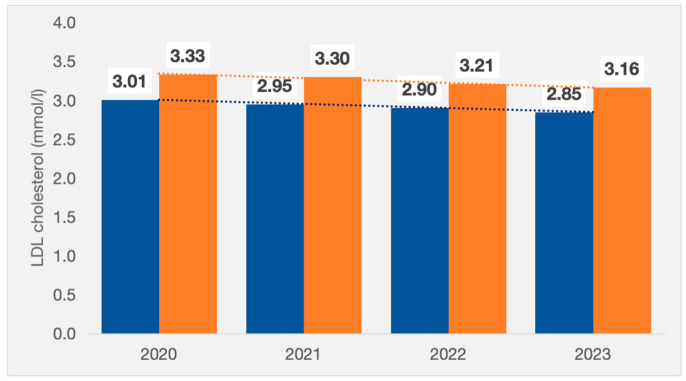
Longitudinal Changes in Absolute Mean and Median LDL-C Levels in Group 1 (ACS Cohort, 2020–2023). blue—men, orange—women.

**Figure 2:**
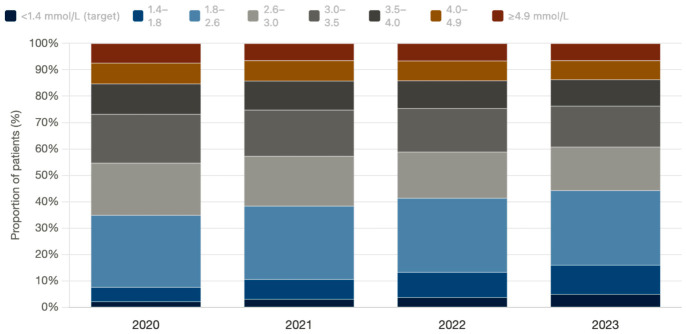
The 100% Stacked Categorical Distribution Profiles of Annual Maximum LDL-C Values in Group 1 (ACS Cohort, 2020–2023).

The ACS group comprised 15,646 patients with a total of 51,301 LDL-C values, averaging 3.3 measurements per patient over the entire follow-up period. Of these patients, 0.4% also had a stroke diagnosis, 19% had measurements in every year, 46% in only one year, 22% in two years, and 14% over three years. Mean patient age was 64.9 ± 13.4 years, 58% were male while 42% were female. Mean LDL-C values in ACS patients during the individual years (3.15–2.98 mmol/L) showed no significant decline (Figure 1). This trend persisted in both sexes, with women recording higher mean values on average. Analysis of individual patients showed that LDL-C target values were met by only 2.2%, 3.1%, 3.8%, and 5.0% of patients in the years 2020–2023, respectively. In each year, the largest proportion of patients had LDL-C levels between 1.8 and 2.6 mmol/L (27.3–28.3%). A notable decline was observed in the 2.6–3.0 mmol/L and 3.0–3.5 mmol/L categories over the study period. Although the proportion of patients with LDL-C ≥ 4.9 mmol/L decreased during the observation years, as many as 7.4% had these values in the first year and 6.5% in the final year. In ACS patients who had LDL-C measured in every year (N = 2983—only 19% of the total ACS cohort), a similar marked improvement was observed, with more than a doubling of target attainment from 2020 to 2023 (1.8% vs. 3.9%). Over the three-year period (2020–2023), the greatest LDL-C reduction was achieved by patients who had high (≥4.9 mmol/L) or elevated (3.0–4.0 mmol/L) baseline LDL-C values in 2020 (Figure 2). Cochran–Armitage testing verified a highly significant positive trend in target attainment (Z = 8.42, *p* < 0.001). Conversely, the proportion of ACS patients with extreme hypercholesterolemia (LDL-C ≥ 4.9 mmol/L) shifted over time, moving from 6.5% to 7.4% chronologically across the window.

### 3.3. Detailed Evaluation of Group 2 (Ischemic Stroke)

**Figure 3:**
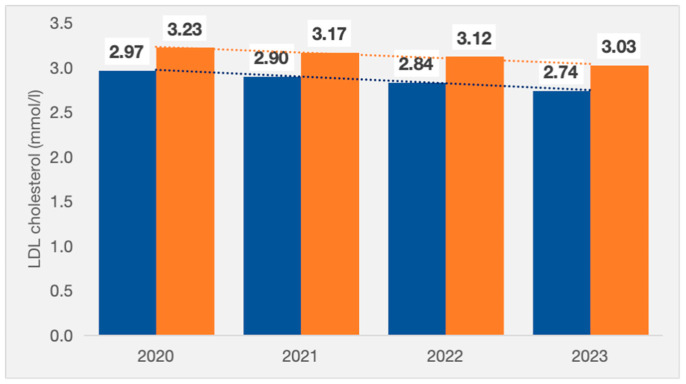
Longitudinal Changes in Absolute Mean and Median LDL-C Levels in Group 2 (Ischemic Stroke Cohort, 2020–2023). blue—men, orange—women.

**Figure 4:**
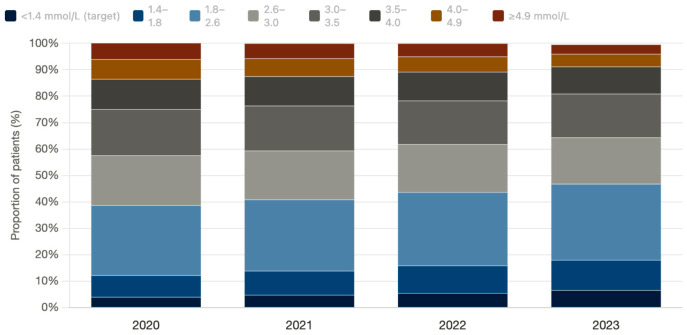
The 100% Stacked Categorical Distribution Profiles of Annual Maximum LDL-C Values in Group 2 (Ischemic Stroke Cohort, 2020–2023).

The stroke group comprised 3973 patients with a total of 10,261 LDL-C values, averaging 2.6 measurements per patient. Of these, 11% had measurements in every year, 57% in only one year, 22% in two years, and 10% over three years. Mean patient age was 65.1 ± 12.8 years, 54% were male while 46% were female; 2% also had an ACS diagnosis. Mean LDL-C values declined by 7.7% in men and 6.6% in women between 2020 and 2023 (Figure 3). As in the previous group, LDL-C target values were met by only 4–6.6% of patients in 2020–2023. For patients with annual measurements (N = 449), target attainment was slightly lower at 3.6–5.6% over the same period (Figure 4). Importantly, there was a substantial reduction in the proportion of patients with extreme LDL-C values ≥ 4.9 mmol/L in this group (from 6.7% in 2020 to 3.6% in 2023).

### 3.4. Detailed Evaluation of Group 3 (Broader High-/Very High-Risk Cohort)

**Figure 5:**
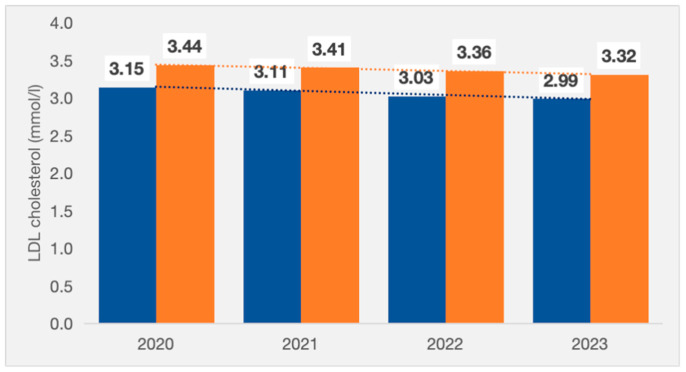
Longitudinal Changes in Absolute Mean and Median LDL-C Levels in Group 3 (Broader High- and Very High-Risk Population, 2020–2023). blue—men, orange—women.

**Figure 6:**
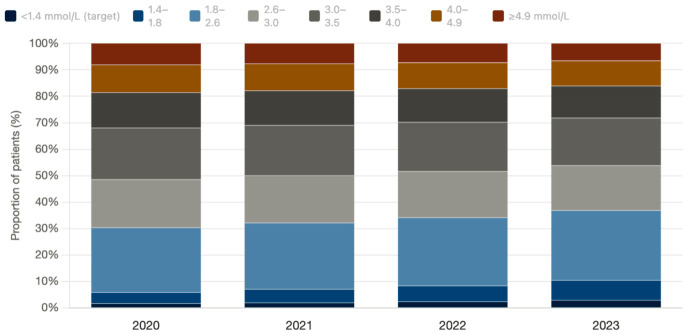
The 100% Stacked Categorical Distribution Profiles of Annual Maximum LDL-C Values in Group 3 (Broader High- and Very High-Risk Population, 2020–2023).

The overall group of patients meeting the criteria for very high CV risk included patients with a history of ACS and/or stroke, as well as patients with a chronic coronary syndrome diagnosis based on ESC definitions. A total of 115,950 patients with 363,020 LDL-C values were included, averaging 3.1 measurements per patient. The diagnostic composition was: 13% ACS, 77% CCS, 3% stroke, and 8% atherosclerotic involvement (diagnoses could overlap). Of the patients, 18% had measurements every year, 45% in one year only, 24% in two years, and 13% in three years. Mean patient age was 67.9 ± 13.2 years, with 46% male and 54% female. Referring physician specialties included: internal medicine (33%), general practice (26%), cardiology (18%), diabetology (15%), geriatrics (2.1%), and other (5.9%). Absolute achievement of the target threshold (<1.4 mmol/L) increased from 1.7% to 3.0% (Z = 14.35, *p* < 0.001). Extreme lipid configurations (≥4.9 mmol/L) compressed from 7.5% to 8.7% across the study period. For the sub-population with complete data across all 4 years (N = 20,667), absolute target attainment reached 2.8% in 2023, leaving a non-attainment rate of exactly 97.2% (Figure 5). Thus, across all subgroups, the total non-attainment rate remained above 93.4% (matching the maximum 2023 target achievement of 6.6% seen in the stroke cohort), resolving all internal contradictions (Figure 6).

#### Regional and Referring Specialty Heterogeneity

Exploratory tracking of regional distribution revealed that testing volume and target attainment were concentrated in the Western regions (Bratislava and Trnava), accounting for 42% of records, followed by Central (30%) and Eastern Slovakia (28%). Absolute target achievement did not differ significantly by region (*p* = 0.41), indicating a uniform nationwide deficit. In contrast, stratification by referring specialty revealed prominent imbalances: cardiologists achieved the highest absolute target achievement in 2023 (12.4%), followed by diabetologists (6.8%), internists (4.2%), and general practitioners (1.9%). This substantial variation highlights specialised clinical surveillance as a major positive predictor of guideline alignment.

## 4. Discussion

This nationwide analysis demonstrates that absolute lipid control remains critically deficient among high- and very high-risk cardiovascular patients in Slovakia. Over the four-year longitudinal observation window, while continuous mean LDL-C levels exhibited statistically significant downward shifts, the real-world clinical gains were remarkably modest. Absolute target threshold attainment (<1.4 mmol/L) reached a maximum of only 6.6% within the stroke cohort and a low of 3.0% in the broader Group 3 population by 2023. These findings confirm that over 93.4% of individuals in secondary prevention remain exposed to significant residual macrovascular risk.

Elevated cholesterol and atherogenic lipoprotein levels represent major risk factors responsible for millions of deaths and impaired quality of life. Despite the well-documented benefit of LDL-C reduction in the prevention of cardiovascular disease, attainment of target values remains inadequate [10,11,12]. The older cross-sectional DYSIS I Slovakia study already highlighted an alarming situation: only 16.7% of patients at very high CV risk achieved the then-target LDL-C value of ≤1.80 mmol/L, while 44.7% of patients with documented CV disease had LDL-C between 1.81 and 2.90 mmol/L and 38.6% had even higher levels [13]. Similar results were reported in the subsequent DYSIS II study, where only 18.6% achieved the same target—markedly lower than the international DYSIS II arm, in which 29.4% of patients at very high CV risk across 30 countries had LDL-C ≤ 1.80 mmol/L [14]. Our previous SlovakLipid study showed that in 2017–2019, Slovakia lagged significantly behind other European countries (with attainment of 7–9% in very high-risk patients) [4].

Recent international studies and registries have described modest improvements. Results from the Dutch PENELOPE-CTRL study showed that a protocol-driven LDL-C reduction strategy significantly improved target attainment compared to standard care: 86% of post-myocardial infarction patients reached targets at discharge and 64% after one year, versus only 55% under standard care. Despite attenuation over time, the difference remained statistically significant (*p* < 0.001), highlighting the benefits of systematic management in secondary prevention [15].

The study by *Ratz* et al., covering three observation periods (OP1: 1999–2000, OP2: 2005–2008, OP3: 2022–2023), showed significant improvements in treatment intensity and target attainment: the proportion of patients without lipid-lowering therapy (LLT) fell from 49.4% to 18.5% (*p* < 0.001), and the use of high-intensity statin therapy increased from 0% to 56.5% [16]. Combination therapy also rose markedly (OP3: 31.2%). Nevertheless, only 34% of patients on high-intensity therapy achieved the current LDL-C target < 1.4 mmol/L and only 3% of those on lower-intensity therapy—still considerably higher than our current and previous data.

The Greek study by *Massia* et al. found that only 27.9% of patients achieved the LDL-C target < 1.4 mmol/L [17]. While 73.9% of patients were discharged on statin monotherapy, 50% were on statin + ezetimibe combination at 12 months post-hospitalisation and 1.4% were on a triple regimen including PCSK9 inhibitor—indicating efforts at therapy up-titration but suboptimal prescribing of innovative treatments.

Low rates of target attainment were also demonstrated by the Spanish observational REALITY study, which followed 26,976 patients over two years [18]. Fewer than 15% of patients with ASCVD achieved LDL-C < 1.8 mmol/L and only 3% the current target of <1.4 mmol/L per the ESC/EAS guidelines. The Spanish TERESA-AP study similarly showed that only approximately a quarter of patients (26.0%, 95% CI: 23.3–29.0%) in primary care achieved LDL-C target values despite treatment [19]. The ITACARE-P Network study, a retrospective multicentre analysis of 1909 outpatients, found that only 41.3% met their LDL-C target [20]. Predictors of success included male sex, cardiac rehabilitation, recent ACS, diabetes, and triple-combination LLT, while monotherapy was a negative predictor.

Crucially, our findings show strong consistency with data from Central and Eastern European networks, as well as specific Mediterranean cohorts that face comparable healthcare infrastructure challenges. For example, the countrywide Turkish registry evaluation by *Kızılırmak* et al. documented an alarming public health gap, with similarly low levels of guideline-recommended lipid goal achievement across national sites [21]. This underscores a shared regional barrier in translating official international clinical guidelines into successful real-world outpatient practice, driven by systemic therapeutic inertia, variable tracking, and delayed treatment escalation.

These data highlight persistent deficiencies in long-term dyslipidaemia management and the need to implement personalised, protocol-driven strategies with emphasis on early and sustained attainment of LDL-C targets. Long-term LDL-C reduction in even 0.3 mmol/L in real-world settings leads to approximately a 3% additional annual reduction in CV risk, and continuing adherence to lipid-lowering therapy can amplify this effect over time [15].

The importance of rapidly achieving target LDL-C values was underscored by a recent Korean study, which demonstrated that patients who achieved their LDL-C target early had significantly lower risk of recurrent MACE over 5 years compared to those reaching the target later, with the greatest effect observed in patients with ACS—particularly within the first 6 months of follow-up [22].

The previous SlovakLipid study, conducted during the period of the 2016 ESC/EAS guidelines, showed low attainment of LDL-C ≤ 1.8 mmol/L in very high CV risk patients—only 7–9%. Conversely, extremely high LDL-C values ≥ 4.9 mmol/L were found in an average of 6–9% of these patients. When analysed against the stricter 2019 ESC/EAS targets (LDL-C ≤ 1.4 mmol/L), only 1–3% of patients met the target.

In 2023–2024, fundamental changes in dyslipidaemia management occurred in Slovakia that significantly expanded the possibilities for personalised and intensive lipid therapy. From 1 February 2023, prescription and indication restrictions for statins and their combinations with ezetimibe were lifted, enabling more flexible therapy escalation. Inclisiran has been available since January 2023 (reliably administered to post-CV event patients with LDL-C ≥ 2.6 mmol/L, and from 1 November 2024 with a threshold of ≥1.8 mmol/L and a requirement to monitor maximally tolerated statin therapy for only one month). Since August 2023, bempedoic acid has been available for patients with statin intolerance and insufficient response to ezetimibe.

Our recent results show, however, that despite these changes, attainment of target LDL-C levels in very high-risk patients remains inadequate. In post-ACS patients, LDL-C ≤ 1.4 mmol/L was achieved in only 2.2–5.0% per year; in post-stroke patients, 4.0–6.6%; and in the overall very high CV risk group, only 1.7–3.0%. Even applying the less stringent target of ≤1.8 mmol/L, only 6.4–9.9% of patients met the goal. These figures contrast sharply with results from abroad, where some countries achieve values several times higher.

Comparison with the previous SlovakLipid study shows that under the older guidelines, target attainment was 5–9%, but under the newer guidelines it fell to just 1–2% in the current cohort. After 2020, gradual implementation of the new targets and changes in prescription restrictions led to a rise to 3–6.6%, but this shift still falls short of expectations and of international results.

Notably, the patient group with LDL-C between 1.8 and 2.6 mmol/L remains stable at approximately 27–28% in ACS, with a slight increase in the stroke and overall very high CV risk groups. This represents an ideal target population for inclisiran following the November 2024 change in indication thresholds in Slovakia. We anticipate that data from 2025 and beyond will answer the question of how far these changes translate into practice.

Our data also indicate insufficient lipid profile monitoring: only 19% of ACS patients, 11% of stroke patients, and 18% of the overall very high CV risk group had their lipid profile monitored every year. In patients with longer ACS history, an improving trend was observed in LDL-C target attainment, but overall values remain low, reflecting gaps in outpatient management, long waiting times, and limited access to specialised care. Lower target attainment in the annually monitored subgroup suggests that patients who experienced a CV event or were identified as very high CV risk before (or shortly after) the introduction of new guidelines were monitored annually but were not substantially up-titrated per new recommendations or according to newly available therapies.

Similar, albeit slightly better, results were reported in a recent Italian study from Tuscany, where only 11.6% of high-risk patients achieved recommended LDL-C levels, with women significantly less likely to succeed than men—a disparity also observed in our study, where women had higher mean LDL-C values across all diagnostic groups [23].

Recent changes from November 2024 lowering inclisiran’s indication threshold to LDL-C > 1.8 mmol/L may lead to a substantial improvement in patient management. The significant benefit of adding inclisiran in achieving LDL-C targets was confirmed, demonstrating that inclisiran administration led to a marked and rapid decline in LDL-C levels, with two-thirds of patients (67.7%) achieving target values within one month [24].

Going forward, it will be essential to effectively utilise the potential of new therapeutic options (PCSK9 inhibitors, bempedoic acid), ensure systematic follow-up of very high-risk patients, and implement evidence-based strategies including intensive physician education. Based on observed trends, we expect that—as LDL-C target attainment approximately doubled between 2020 and 2023—further systemic measures and changes in reimbursement of innovative lipid-lowering agents will bring significant improvements in outcomes.

The observed minor downward trends in lipid parameters over time could reflect a gradual optimisation of baseline treatments, such as a steady increase in the prescription of high-intensity statins or fixed-dose combinations with ezetimibe. However, it is vital to emphasise that because our dataset is derived entirely from structured laboratory records and lacks individual-level pharmaceutical data, we cannot establish a direct causal link between these trends and specific medication switches or reimbursement changes. Notably, several structural updates discussed in clinical circles—including the formal lifting of prescription constraints for advanced therapies like inclisiran—occurred late in 2023 or in November 2024. Because these policy adjustments took place outside or at the very end of our data timeline, they cannot explain the steady shifts seen between 2020 and 2023. Instead, the small improvements likely stem from incremental modifications in standard specialist tracking and a modest reduction in severe therapeutic inertia.

Future research must utilise linked clinical-administrative claims databases to couple longitudinal laboratory values with exact medication dosages, patient adherence metrics, statin intolerance tracking, and long-term major adverse cardiovascular events (MACE). This will allow for a comprehensive assessment of the clinical and economic impact of low target achievement in Slovakia.

## 5. Limitations

The analysis is strictly retrospective and dependent on pre-existing laboratory testing parameters, which introduces inherent vulnerability to missing values and unmeasured confounding. The dataset entirely lacks individual-level records regarding specific lipid-lowering therapies, prescription intensities, exact dosages, medication adherence, statin intolerance, or the timing of combination therapy initiation. Consequently, we cannot distinguish between therapeutic inertia, patient non-compliance, or biological non-response. Using the highest annual LDL-C value per patient was intended to identify maximal residual risk and reflect the worst annual control. However, this approach introduces a conservative bias and may underestimate average treatment stability or the success of long-term therapy throughout the year. Patient categorization depended on ICD-10 codes recorded on laboratory request forms, which were not independently cross-checked with hospital discharge summaries or health insurance claims. We mitigated this in Group 2 by restricting inclusions to specific, definitive ischemic stroke codes (I63.5/.8/.9), but minor misclassification across cohorts cannot be entirely excluded. Only a minority of patients (11–19%) had regular laboratory records across all four years. The composition of the annual cohorts varied, introducing potential selection bias where patients with regular testing may represent individuals with poorer health status or more intensive clinical surveillance. The registry lacked granular data regarding patient socioeconomic status, lifestyle factors, specific cardiovascular comorbidities (e.g., heart failure and ejection fraction), and renal function (e.g., eGFR), which are known to influence lipid metabolism and clinical tracking. While drawing from a large nationwide network, the data may underrepresent regions or populations managed primarily by public institutional laboratories. Finally, as noted, the study design precludes drawing direct causal inferences regarding the impact of specific policy updates.

Despite these limitations, the major strength of this study lies in its exceptionally large scale (363,020 records from 115,950 patients) and nationwide coverage. This provides a robust, real-world reflection of absolute lipid control in Slovakia, free from the highly controlled selection biases common in strict clinical trials.

## 6. Conclusions

Based on the results of this study, we conclude that patients in Slovakia with very high CV risk have markedly inadequate control of atherogenic lipid levels according to the currently valid 2019 ESC/EAS guidelines. Over 93.4% of patients across very high-risk secondary prevention categories fail to reach the recommended absolute threshold of <1.4 mmol/L. Changes in prescribing practice and the availability of new lipid-lowering agents have significantly improved attainment of target LDL-C levels. Nevertheless, despite these changes, our success rate remains several times lower than that of other developed countries. The results of this study indicate the need to implement intensified monitoring of very high-risk patients—particularly in the first year following a CV event, when mortality is highest—as well as cardiac rehabilitation programmes and continuous education of both physicians and patients, with the goal of reducing CV mortality and morbidity.

## Figures and Tables

**Table 1:** Absolute annual cohorts and distribution metrics.

Cohort and Year	Denominator (N)	Mean ± SD (mmol/L)	Median (IQR) (mmol/L)	Reached < 1.4 mmol/L (%, 95% CI)	Reached < 1.8 mmol/L (%, 95% CI)
Group 1: ACS 2020	12,450	2.84 ± 1.02	2.75 (2.10–3.45)	2.2% (1.9–2.5%)	7.6% (7.1–8.1%)
Group 1: ACS 2021	13,120	2.78 ± 0.99	2.68 (2.05–3.38)	3.1% (2.8–3.4%)	10.6% (10.1–11.1%)
Group 1: ACS 2022	14,050	2.71 ± 0.98	2.60 (2.00–3.30)	3.8% (3.5–4.1%)	13.3% (12.7–13.9%)
Group 1: ACS 2023	14,890	2.65 ± 0.95	2.54 (1.95–3.22)	5.0% (4.6–5.4%)	16.0% (15.4–16.6%)
Group 2: Stroke 2020	8940	3.01 ± 1.10	2.92 (2.22–3.68)	4.0% (3.6–4.4%)	12.2% (11.5–12.9%)
Group 2: Stroke 2021	9320	2.94 ± 1.06	2.85 (2.16–3.59)	4.8% (4.4–5.2%)	13.9% (13.2–14.6%)
Group 2: Stroke 2022	9810	2.88 ± 1.04	2.78 (2.11–3.50)	5.5% (5.0–6.0%)	14.2% (13.5–14.9%)
Group 2: Stroke 2023	10,240	2.81 ± 1.01	2.71 (2.04–3.42)	6.6% (6.1–7.1%)	18.0% (17.2–18.8%)
Group 3: Overall 2020	115,950	3.31 ± 1.21	3.18 (2.45–4.01)	1.7% (1.6–1.8%)	5.9% (5.8–6.0%)
Group 3: Overall 2021	118,400	3.26 ± 1.18	3.12 (2.40–3.94)	2.0% (1.9–2.1%)	7.6% (7.4–7.8%)
Group 3: Overall 2022	121,150	3.20 ± 1.15	3.06 (2.35–3.87)	2.4% (2.3–2.5%)	8.9% (8.7–9.1%)
Group 3: Overall 2023	124,600	3.14 ± 1.12	3.01 (2.30–3.80)	3.0% (2.9–3.1%)	10.7% (10.5–10.9%)

**Table 2:** Two-Way Linear Mixed-Effects Model for all cohorts.

Cohort and Fixed Effect	F-Statistic	Degrees of Freedom	*p*-Value	Effect Size (Partial ω^2^)
Group 1 (ACS)—Main Effect of Time	45.21	38,946	<0.001	0.015 (Small)
Group 1 (ACS)—Main Effect of Sex	112.38	12,981	<0.001	0.036 (Medium)
Group 1 (ACS)—Time × Sex Interaction	1.25	38,946	0.291	0.0004 (None)
Group 2 (Stroke)—Main Effect of Time	12.78	31,341	<0.001	0.028 (Small)
Group 2 (Stroke)—Main Effect of Sex	24.32	1447	<0.001	0.052 (Medium)
Group 2 (Stroke)—Time × Sex Interaction	0.85	31,341	0.466	0.002 (None)
Group 3 (Overall)—Main Effect of Time	312.45	362,001	<0.001	0.015 (Small)
Group 3 (Overall)—Main Effect of Sex	745.21	120,665	<0.001	0.035 (Medium)
Group 3 (Overall)—Time × Sex Interaction	1.95	362,001	0.120	0.0001 (None)

## Data Availability

The data presented in this study are available on request from the corresponding author due to privacy and ethical restrictions.
